# Dual-Achilles Reconstruction for Chronic Quadriceps Rupture (DARC) Technique as a Novel Surgical Technique for Chronic Quadriceps Tendon Rupture: A Case Report

**DOI:** 10.7759/cureus.99937

**Published:** 2025-12-23

**Authors:** Kamil R Jarjess, John E Tobia, Aidan M Brikho, Rafe Affas, Jamil Haddad, Matthew Yousif

**Affiliations:** 1 College of Arts and Sciences, Oakland University, Rochester, USA; 2 College of Natural Sciences, Michigan State University, East Lansing, USA; 3 Department of Osteopathic Medicine, Touro College of Osteopathic Medicine, Middletown, USA; 4 Lyman Briggs College, Michigan State University, East Lansing, USA; 5 Department of Orthopedic Surgery, McLaren Macomb Hospital, Mount Clemens, USA; 6 Department of Orthopedic Surgery, Corewell Health William Beaumont University Hospital, Royal Oak, USA

**Keywords:** achilles allograft, chronic quadriceps ruptures, extensor lag, quadriceps tendon injury, quadriceps tendons rupture

## Abstract

Quadriceps tendon ruptures (QTRs) are uncommon injuries that can become challenging to manage when diagnosis is delayed and significant tendon retraction and scarring occur. In chronic cases with significant tendon defects, primary repair is often not feasible, and reconstruction with graft augmentation becomes necessary. Achilles tendon allografts are commonly used in such scenarios; however, obtaining reliable graft incorporation and stable load-sharing remains difficult in patients with large defects, poor tendon quality, or elevated mechanical demand. This report describes a novel dual-layer Achilles tendon allograft reconstruction, referred to as the Dual-Achilles Reconstruction for Chronic Quadriceps Rupture (DARC) technique. This technique is designed to maximize circumferential graft contact, restore tendon bulk, and improve load distribution in a chronic QTR with significant retraction.

A 56-year-old man with a body mass index (BMI) of 42.2 presented six weeks after two mechanical falls with persistent pain, recurrent buckling, and loss of active knee extension. Examination revealed a palpable suprapatellar defect, patella baja, and complete extensor mechanism failure. Radiographs demonstrated a Caton-Deschamps index of 0.56, and magnetic resonance imaging confirmed a full-thickness QTR with approximately 8 cm of proximal retraction and a 7 mm hematoma. Because of the chronicity of the injury and poor-quality tendon tissue, primary repair was not suitable. Reconstruction was performed using two Achilles tendon allografts anchored to the medial and lateral thirds of the superior patellar pole, which were then passed through the quadriceps muscle belly. The grafts were circumferentially wrapped around the tendon stump to create an anterior-posterior dual-layer construct intended to enhance graft integration and biomechanical stability. The patient followed a structured rehabilitation protocol.

The patient regained full knee extension without extensor lag, achieved 0-120° of flexion, normalized gait by 12 weeks, and returned to unrestricted activity by one year. This favorable recovery is notable given the chronic nature of the rupture, the 8 cm retraction, and the patient's high BMI, all of which typically predict poorer outcomes.

This case suggests that dual-layer Achilles tendon allograft reconstruction may offer a viable and structurally robust alternative for chronic QTRs with significant tissue loss where direct repair or single-graft techniques may be insufficient. Further investigation in larger patient cohorts is needed to evaluate the reproducibility, long-term performance, and broader applicability of this technique.

## Introduction

The quadriceps muscle group, consisting of the vastus intermedius, vastus lateralis, vastus medialis, and rectus femoris, serves as the primary agonist responsible for knee extension [1,2]. Working together with the patella and patellar tendon, the quadriceps muscles generate the concentric force that drives the extensor mechanism, functioning in coordination with the hamstrings and surrounding thigh musculature to maintain stability of the knee and lower leg [1,2]. Due to its role in withstanding substantial tensile loads and assisting in active knee extension, rupture of the quadriceps tendon can compromise force transmission through the extensor mechanism, leading to marked functional impairment and weakness in active extension, often manifesting clinically as extensor lag, where active knee extension fails to reach the full passive range [3-5]. Subsequently, in cases of rupture, as the distance between the ruptured quadriceps tendon and the patella increases, patellar height can be compromised, resulting in patellar baja, a condition in which the patella sits abnormally low relative to the trochlear groove and contributes to reduced extensor mechanism efficacy [4,6]. Although patella baja is often reported as a complication of chronic quadriceps tendon rupture (QTR), patellar height can vary naturally among individuals. A recent population-based analysis of 434 healthy knees demonstrated that conventional diagnostic cutoffs frequently misclassify normal knees as having patella baja, indicating that low patellar height may represent a normal anatomical variant rather than a pathologic finding [6,7].

QTRs are a rare injury that occurs in approximately 1.37 per 100,000 persons, disrupting the knee's extensor mechanism by impairing active knee extension [2,8]. Upon physical examination, patients with QTRs typically present with difficulties extending their knee, extensor lag, and other palpable defects in the suprapatellar region [9]. Alongside the clinical examination of QTRs, imaging modalities such as ultrasound and magnetic resonance imaging (MRI) are also vital for confirming tendon discontinuity, assessing retraction distance, differentiating the degree of rupture, and accurate diagnosis [10,11]. 

QTRs are most often linked to direct or indirect trauma, such as simple falls, with several cases reporting the knee in flexion at the time of impact [2,8]. There have also been associations between QTR and systemic comorbidities, such as rheumatoid diseases, chronic renal failure, obesity, diabetes mellitus, as well as the use of anabolic steroids and medications including corticosteroids and fluoroquinolones [8,12]. QTRs occur most in middle-aged men, with a mean age of 51.1, and show a male predominance with a 4.2:1 male-to-female ratio [13]. 

Although QTRs usually impair active knee extension, the degree of limitation depends on the severity of tendon damage [2]. In some cases, compensatory activation of the patellar retinaculum or iliotibial band allows partial extension, contributing to frequent misdiagnosis [14]. QTR patients are often fall-prone due to impaired extension and frequently stabilize themselves by keeping the injured leg straight [14,15]. 

In order to optimize QTR treatment, early diagnosis at the initial stage of rupture is vital [16]. Early surgical management of QTR is recommended to optimize clinical outcomes, as delays can lead to tendon retraction, increased surgical difficulty, and reduced functional recovery [2,14,17]. QTRs are considered chronic when treatment is delayed beyond 3-6 weeks, at which point the injury transitions out of the acute and subacute phases [4]. In cases of acute QTR, the literature indicates that primary repair techniques utilizing transosseous patellar sutures or suture anchor alternatives are considered the standard operative protocol [18,19]. 

In contrast, chronic QTRs are generally not feasible for primary repair techniques due to the possibility of the tendon being retracted or enveloped in scar tissue [20]. Consequently, existing literature supports several reconstruction options for chronic QTRs, including synthetic mesh/scaffolds augmentation, V-Y lengthening, tendon autografts, and Achilles tendon calcaneal bone block allografts as reliable repair strategies [13,20-23]. Advances in graft technology have led to the increased use of Achilles tendon bone-tendon allografts, which offer shorter operating times and comparable biomechanical strength and elasticity to autografts [20]. However, in patients with chronic QTRs and large tendon gaps, achieving optimal graft incorporation and balanced load distribution can be challenging due to retraction, atrophy, and poor tissue quality [24].

To our knowledge, we present a novel technique (Dual-Achilles Reconstruction for Chronic Quadriceps Rupture (DARC)) that circumferentially envelops the native quadriceps tendon, increasing the graft contact surface area and load-sharing. This report describes the surgical technique and postoperative outcome in a 56-year-old male patient with a body mass index (BMI) of 42.2 and a chronic ~8 cm quadriceps tendon defect. 

## Case presentation

A 56-year-old man with a BMI of 42.2, no prior quadriceps tendon pathology, and no significant past medical history presented to the clinic six weeks following two mechanical falls, first from a flight of stairs and subsequently from countertop height one week later. He reported persistent right knee pain, repeated buckling during ambulation, and inability to actively extend the right leg. On physical examination, the patient demonstrated a palpable defect proximal to the patella, a lack of active extension, and diminished quadriceps tone. Passive range of motion was preserved, but there was a complete loss of extensor mechanism function. 

**Figure 1:**
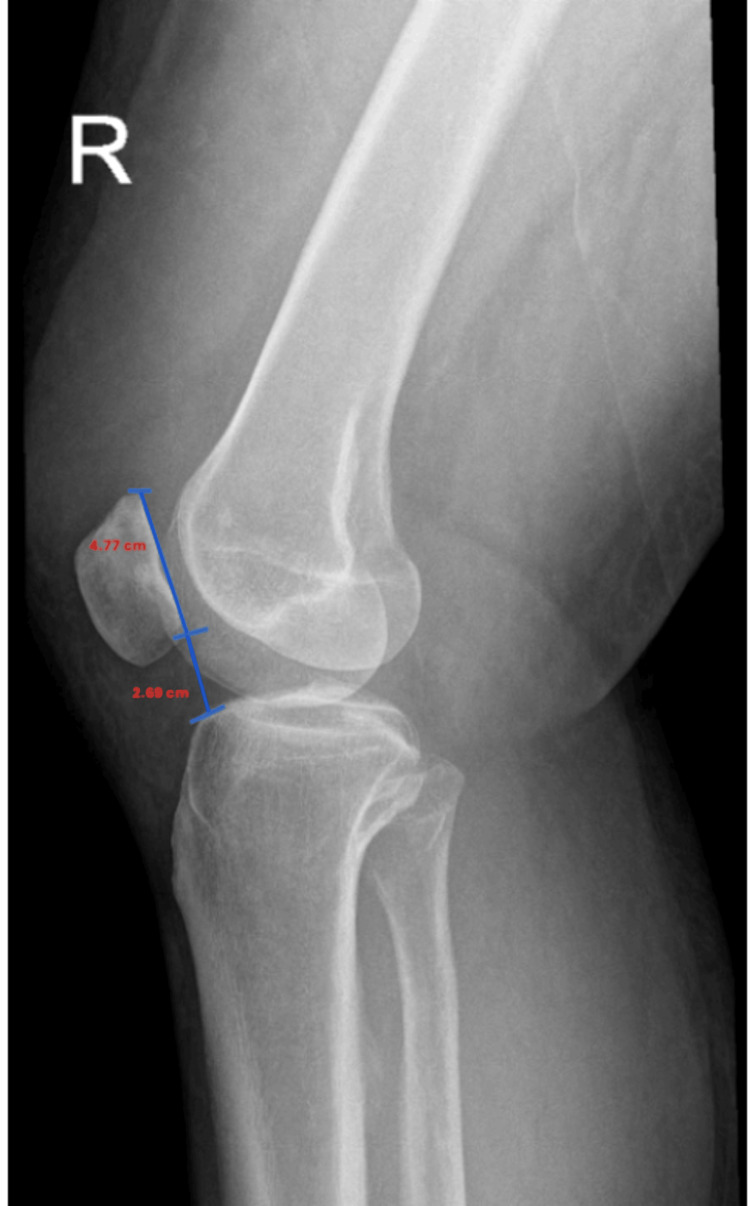
Lateral knee radiograph demonstrating CDI measurement Lateral radiograph of the right knee showing the CDI calculation with a patellar articular length of 4.77 cm and patellar tendon length of 2.69 cm (CDI=0.56). CDI: Caton-Deschamps Index

Initial radiographic imaging included a lateral knee radiograph, which demonstrated a Caton-Deschamps Index (CDI) of 0.56, consistent with patella baja (Figure 1).

**Figure 2:**
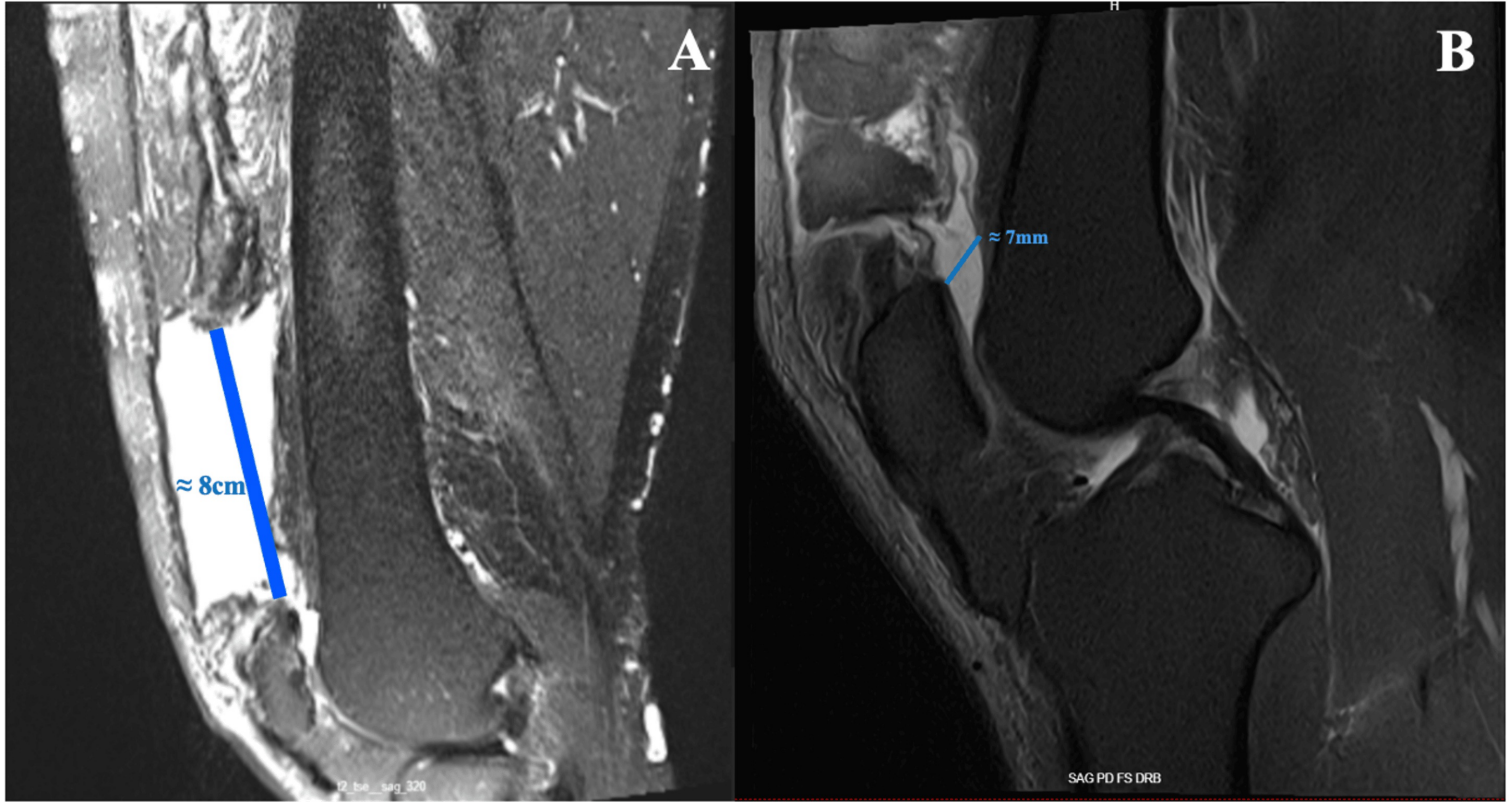
Preoperative sagittal MR images. T2-weighted sequence (A) demonstrates severe proximal quadriceps tendon retraction measuring approximately 8 cm. Proton density fat-suppressed sequence (B) highlights a ~7 mm hematoma within the residual tendon gap MR: magnetic resonance

MRI of the right knee revealed a complete distal QTR with approximately 8 cm of proximal tendon retraction and a 7 mm hematoma filling the resultant defect (Figure 2A-2B).

Given the chronicity of the tear, the degree of retraction, and the poor quality of the residual tendon, direct repair was deemed unfeasible. Surgical reconstruction with dual Achilles tendon allografts was planned. The patient was informed of the risks, benefits, and alternatives and provided written informed consent for both the surgical intervention and inclusion of intraoperative photographs in this report.

**Figure 3:**
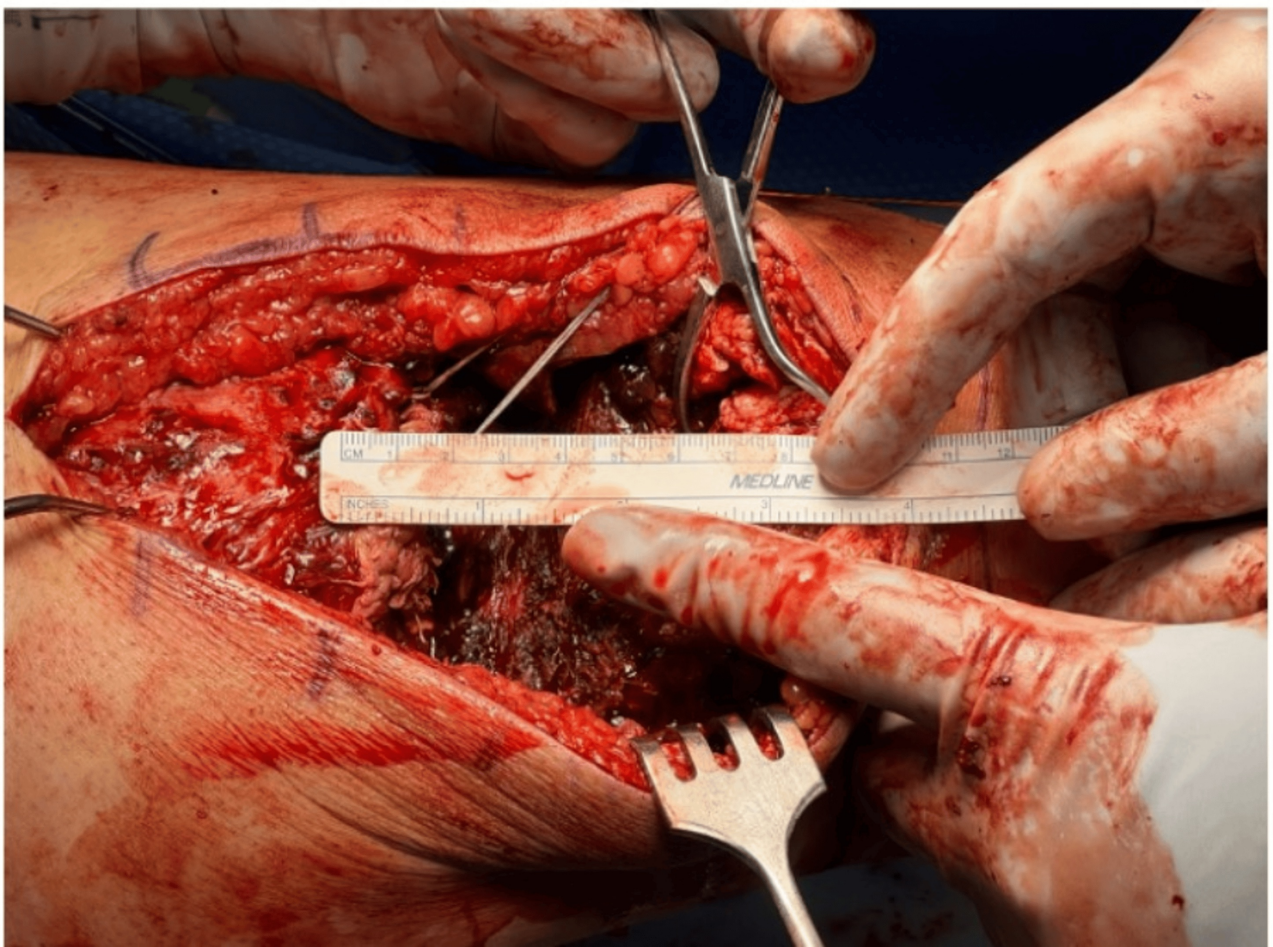
Intraoperative assessment of the chronic quadriceps tendon rupture showing an ~8 cm proximal retraction from the superior patellar pole, confirming the need for allograft reconstruction

A midline longitudinal incision was made from the distal third of the thigh to the inferior pole of the patella. Blunt dissection was carried down to identify the proximal quadriceps tendon, which demonstrated an 8 cm gap to the superior pole of the patella (Figure 3).

**Figure 4:**
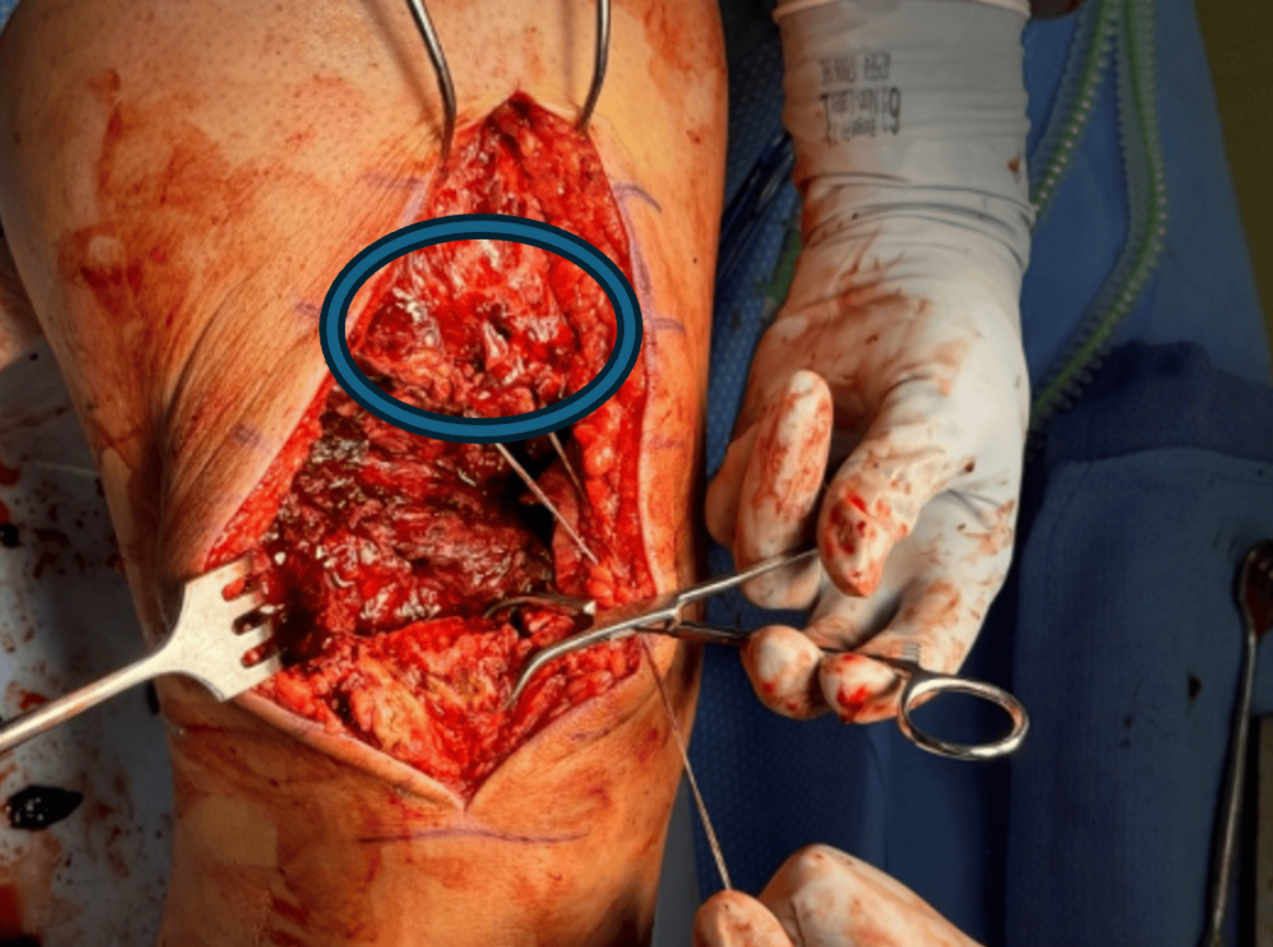
Intraoperative view demonstrating the chronically retracted quadriceps tendon stump (blue circle) surrounded by dense scar tissue and adhesions. These adhesions required careful mobilization to restore tendon length and prepare the native stump for reconstruction with dual Achilles allografts

Adhesions along the anterior and posterior tendon borders were mobilized using a Cobb elevator and sharp dissection, aided by traction through placed tag sutures (Figure 4).

A slit incision was created longitudinally through the mid‑substance of the native quadriceps tendon. Each Achilles allograft (19.5 cm in length) was shuttled proximally through the quadriceps tendon and further tunneled through the quadriceps muscle belly in a deep‑to‑superficial manner. Two intramuscular channels were established using a hemostat introduced approximately 4 cm proximal to the distal edge of the defect in regions of robust muscle tissue, followed by dilation with a Penrose drain. The medial channel was created at the junction of the medial and middle thirds of the quadriceps muscle and the lateral channel at the junction of the middle and lateral thirds. Each allograft was then passed through its respective channel.

Once delivered proximally, both grafts were tensioned by applying 10-15 lbs of axial traction to achieve equal length and eliminate slack. Tensioning and patellar height assessment were performed with the knee positioned at approximately 40° of flexion. The superior pole of the patella was marked on the grafts to confirm appropriate patellar height, and excess graft length was trimmed before Krackow sutures were placed in each distal limb.

**Figure 5:**
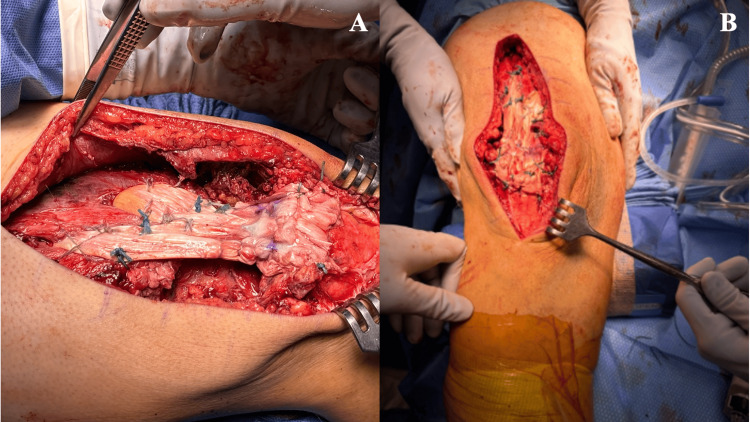
Intraoperative demonstration of the DARC technique. (A) The two Achilles allografts are passed through and circumferentially envelop the native quadriceps tendon and muscle belly. (B) Final construct showing the restored tendon continuity and graft incorporation before closure DARC: Dual-Achilles Reconstruction for Chronic Quadriceps Rupture

The grafts were anchored to the superior pole of the patella using 4.75 mm SwiveLock anchors. Proximally, the grafts were secured to the quadriceps muscle using #2 Ethibond sutures in a spot‑weld pattern to provide circumferential muscle capture and maximize surface contact for biological incorporation. The medial and lateral graft limbs were then sutured together centrally to close the residual tendon gap and reinforce the reconstruction. Approximately 1 cm of additional graft was preserved and sutured into the patellar peritenon for supplemental fixation (Figure 5A-5B). The final construct demonstrated symmetric graft tension, restored patellar height, and appropriate alignment.

**Figure 6:**
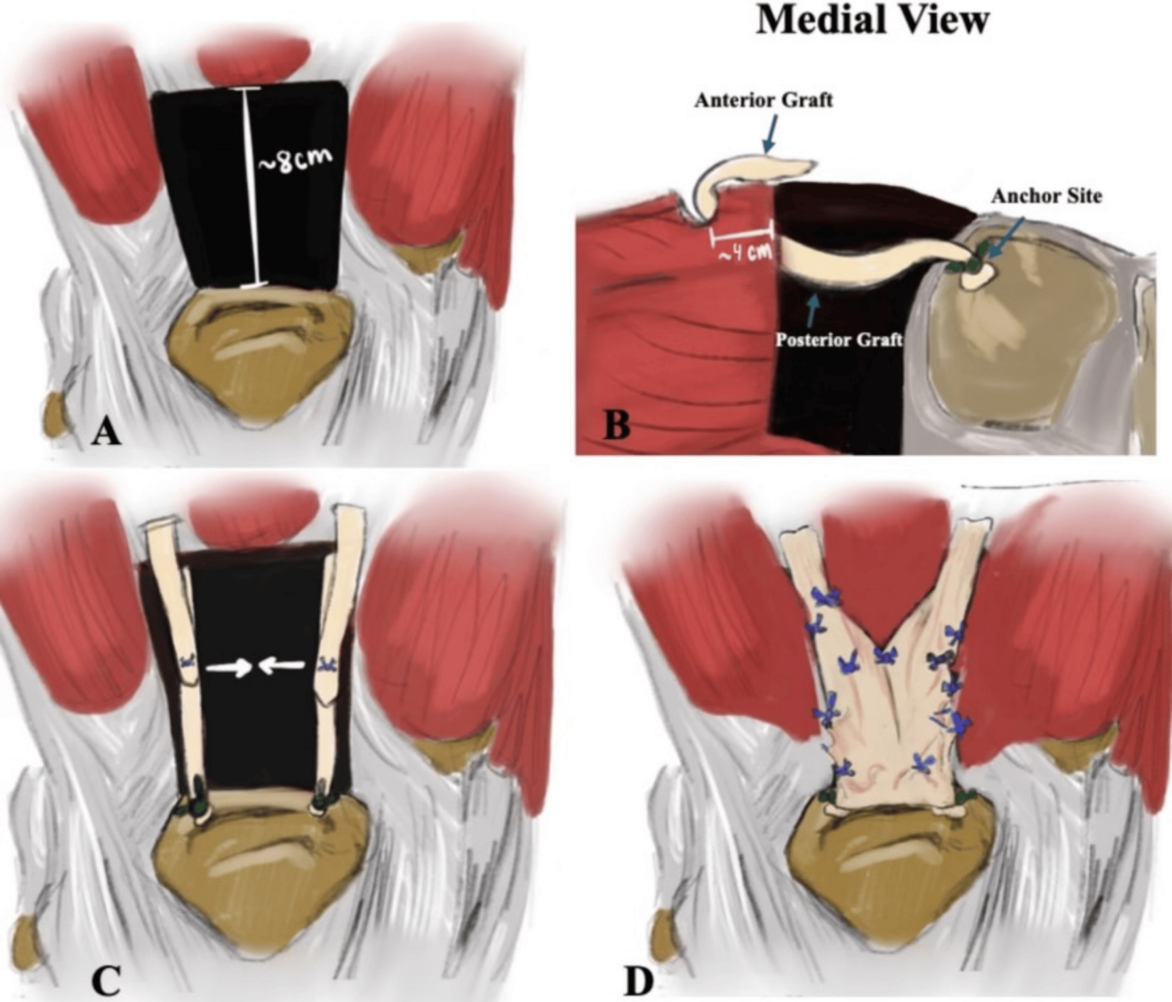
Schematic illustration of the DARC technique (A) Chronic quadriceps tendon rupture demonstrating approximately 8 cm of proximal retraction. (B) Achilles tendon allograft anchored to the superior patellar pole and passed deep-to-superficial through the quadriceps muscle belly approximately 4 cm proximal to the tendon remnant. (C) Medial and lateral Achilles allograft limbs positioned alongside the native tendon after anchor fixation, preparing for circumferential wrapping. (D) Final dual-layer construct with anterior and posterior graft limbs sutured together in a circumferential configuration to maximize contact surface area and load-sharing around the tendon stump. DARC: Dual-Achilles Reconstruction for Chronic Quadriceps Rupture Image Credits: Illustration created by Isabel Maryam Abdal specifically for this case report. Reproduced with permission from the artist.

A stepwise schematic representation of the DARC technique, including graft anchoring, passage, and the final circumferential construct, is shown in Figure 6.

Postoperative rehabilitation course

The patient was placed in a hinged knee brace locked at 15° of flexion immediately postoperatively and instructed to remain toe-touch weight-bearing (TTWB) with the use of crutches. The brace was maintained for a total of six weeks, with removal permitted only for hygiene purposes after the incision had healed. Early rehabilitation included quadriceps, hamstring, and gluteal isometric exercises performed twice daily, ankle pumps, edema control modalities (such as ice, compression, and elevation), and neuromuscular stimulation.

At postoperative week 3, the brace was locked in full extension, and active range of motion (AROM) was initiated from 0° to 45°, progressing by 15° weekly until full flexion was achieved by week 6. The patient advanced to weight-bearing as tolerated (WBAT) by the end of this phase. A stationary bike without resistance was introduced once 95-105° of flexion was reached.

By week 7, closed-chain exercises (mini-squats, heel raises, leg press to 60°) and progressive resistance on the stationary bike were incorporated. Crutches were discontinued once the gait pattern normalized. The brace was discontinued when the patient could perform a straight leg raise without extensor lag.

Between weeks 9 and 12, the patient initiated open-chain quadriceps strengthening (short arc quads, straight leg raises), isokinetic training, and proprioceptive exercises, including single-leg balance drills. At the 12-week mark, the patient demonstrated full knee extension without lag and 0-120° of flexion and ambulated independently with a normalized gait. No signs of graft failure or wound complications were observed.

By six months postoperatively, the patient had regained symmetric quadriceps strength compared to the contralateral limb, resumed low-impact recreational activities, and reported no residual pain or instability.

At one year, the patient demonstrated full active extension, 110° of flexion, a negative extensor lag test, normal gait mechanics, and stable patellar tracking. There were no wound or graft-related complications. Functional performance returned to pre-injury levels without restrictions. Quantitative outcome measures included the following: Quadriceps Index: 99% and International Knee Documentation Committee (IKDC) subjective score: 89/100. This recovery profile supports the structural integrity of the dual-layer Achilles allograft construct and its ability to tolerate early controlled loading.

## Discussion

QTRs are relatively uncommon injuries that are frequently missed at initial presentation, and delayed diagnosis can lead to profound functional impairment when not identified and treated promptly [2,16]. In chronic QTR cases, progressive tendon retraction, scarring, and muscle atrophy can result in poor tendon quality and large gaps that make primary repairs difficult or infeasible [4,20,24]. Our patient presented six weeks after injury with approximately 8 cm of proximal tendon retraction, poor-quality tendon tissue, patella baja, and a BMI of 42.2. These findings represent factors that increase mechanical demand on the extensor mechanism, and in the setting of chronic QTR, such conditions make simple primary repair biomechanically unfavorable. This is consistent with broader extensor mechanism literature showing that reinforced or augmented reconstruction is typically required when tissue quality or load demands are high [20,25].

Although V-Y quadricepsplasty (Codivilla lengthening) has been described for chronic QTR, its use is limited to moderate defects (≤5 cm), with larger gaps considered a contraindication [26]. When tendon loss is significant or the tissue quality is compromised, the literature supports transitioning to augmented or reconstructive techniques rather than attempting lengthening or primary repair alone [25]. Single Achilles tendon allograft reconstruction can be successful; however, outcomes depend on the condition of the residual tendon and achieving proper graft tension, and chronic cases may be at risk for graft elongation and extensor lag [27,28].

The technique utilized in this case was designed to address these specific limitations. The dual-layer Achilles tendon allograft construct allows the native quadriceps tendon remnant to be encased between two broad biologic graft surfaces, one positioned posterior to the tendon stump and one anterior. This configuration increases the surface area available for biologic incorporation, distributes load across a larger footprint, and more closely reproduces the natural thickness and contour of the native quadriceps tendon. Anchoring each graft to the medial and lateral thirds of the superior patellar pole recreates the broad patellar insertion of the native quadriceps mechanism and helps maintain patellar tracking. The proximal "spot-weld" suture pattern used to secure the grafts provides circumferential tissue capture and avoids stress concentrations that can occur with end-to-end suturing alone.

The patient's postoperative progression supports the biomechanical validity of this construct. Our patient regained active knee extension without lag and returned to full ambulation with normalized gait by 12 weeks. At a later follow-up, he demonstrated symmetric quadriceps activation and reported no functional limitations in daily activity. This is noteworthy given his elevated BMI, degree of retraction, and chronic presentation, all of which are factors that typically predict slower or less complete recovery [4,16,29]. The ability to restore full extension without lag suggests that the dual-layer construct provided sufficient immediate stability to withstand early controlled rehabilitation, while the layered graft arrangement likely supported progressive biologic remodeling over time.

Our case showcases the importance of matching reconstructive strategy to defect size, tissue quality, and patient-specific biomechanical demand. In patients with severe tendon loss or large retraction distances, especially those with elevated BMI, constructs that maximize graft contact area and load-sharing may provide a meaningful clinical advantage. While longer-term follow-up and comparative studies are needed, this technique may offer a reproducible and structurally sound alternative for chronic QTR where direct repair or single-graft reconstruction may be insufficient.

## Conclusions

Chronic QTRs with large retraction distances remain difficult to manage, particularly in patients with elevated BMI and poor native tendon quality. This case demonstrates that a dual-layer Achilles tendon allograft construct can restore extensor mechanism integrity, provide immediate stability for early rehabilitation, and achieve excellent functional recovery at one year. By circumferentially enveloping the residual tendon, the DARC technique maximizes graft contact area and load-sharing, offering a structurally robust alternative when primary repair or single-graft reconstruction may be insufficient. Further clinical experience and comparative studies will be necessary to determine the broader applicability and long-term durability of this reconstructive strategy.

## References

[1] Yuan H, Kim MK (2025). Neuromuscular dynamics during isometric knee contractions: effects of target force, knee angle, and tibial rotation on force steadiness. Sci Rep.

[2] Pope JD, El Bitar Y, Mabrouk A (2023). Quadriceps tendon rupture. StatPearls [Internet].

[3] Tandogan RN, Terzi E, Gomez-Barrena E, Violante B, Kayaalp A (2022). Extensor mechanism ruptures. EFORT Open Rev.

[4] Watson SL, Kingham YE, Patel RM (2022). Chronic quadriceps tendon ruptures: primary repair of quadriceps via bioaugmentation and patellar tendon lengthening. Arthrosc Tech.

[5] Kumar V, Kumar K, Arora S (2022). Knee extension lag. Int J Health Sci.

[6] Hockings M, Cameron JC (2004). Patella baja following chronic quadriceps tendon rupture. Knee.

[7] Vella-Baldacchino M, Cipolla A, Asghar Z, LiArno S, Faizan A, Argenson JN, Ollivier M (2025). Patella height ratios diagnose the same healthy knees differently. Sci Rep.

[8] Loose K, Rudolph J, Schlösser M (2023). Quadriceps tendon ruptures in middle-aged to older patients: a retrospective study on the preoperative MRI injury patterns and mid-term patient-reported outcome measures. J Pers Med.

[9] Whitmore JA, Lele P, Lyons JG, Froehle A (2025). Quadriceps tendon ruptures: a narrative review. Ann Jt.

[10] Secko M, Diaz M, Paladino L (2011). Ultrasound diagnosis of quadriceps tendon tear in an uncooperative patient. J Emerg Trauma Shock.

[11] Falkowski AL, Jacobson JA, Hirschmann MT, Kalia V (2021). MR imaging of the quadriceps femoris tendon: distal tear characterization and clinical significance of rupture types. Eur Radiol.

[12] Matthies NF, Paul RA, Dwyer T, Chahal J, Whelan D (2022). A survey of treatment trends for acute quadriceps tendon ruptures among North American surgeons. Orthop J Sports Med.

[13] Oliva F, Marsilio E, Migliorini F, Maffulli N (2021). Complex ruptures of the quadriceps tendon: a systematic review of surgical procedures and outcomes. J Orthop Surg Res.

[14] Jolles BM, Garofalo R, Gillain L, Schizas C (2007). A new clinical test in diagnosing quadriceps tendon rupture. Ann R Coll Surg Engl.

[15] Ponziani L, Tentoni F, Di Caprio F (2022). Quadriceps or patellar ligament reconstruction with artificial ligament after total knee replacement. Acta Biomed.

[16] Duthon VB, Fritschy D (2011). Knee extensor mechanism ruptures [Article in French]. Rev Med Suisse.

[17] Pocock CA, Trikha SP, Bell JS (2008). Delayed reconstruction of a quadriceps tendon. Clin Orthop Relat Res.

[18] Arnold EP, Sedgewick JA, Wortman RJ, Stamm MA, Mulcahey MK (2022). Acute quadriceps tendon rupture: presentation, diagnosis, and management. JBJS Rev.

[19] Brossard P, Le Roux G, Vasse B (2017). Acute quadriceps tendon rupture repaired by suture anchors: outcomes at 7 years' follow-up in 25 cases. Orthop Traumatol Surg Res.

[20] Miu CA, Hurmuz M, Miu LO, Ceachir D, Tatu RF (2025). Reconstruction of chronic quadriceps and Achilles tendon ruptures using Achilles allografts: clinical findings and review of literature. Biomedicines.

[21] Hoang V, Anthony T, Quattrocelli M, Farina E, Meter J, Lattermann C (2023). Quadriceps tendon reconstruction with Achilles tendon bone block allograft. Arthrosc Tech.

[22] Dandu N, Trasolini NA, DeFroda SF, Holland T, Yanke AB (2021). Revision quadriceps tendon repair with bone-Achilles allograft augmentation. Video J Sports Med.

[23] Halvorson RT, Dilallo M, Garcia-Lopez E, Colyvas N, Wong SE (2023). Extensor mechanism reconstruction using Achilles tendon allograft with suture tape augmentation. Arthrosc Tech.

[24] Oliva F, Marsilio E, Migliorini F, Maffulli N (2023). Chronic quadriceps tendon rupture: quadriceps tendon reconstruction using ipsilateral semitendinosus tendon graft. J Orthop Surg Res.

[25] Anderson JT, McLeod CB, Anderson LA (2023). Extensor mechanism disruption remains a challenging problem. J Arthroplasty.

[26] Petersen W, Mustafa HA, Buitenhuis J, Braun K, Häner M (2025). VY-plasty for chronic quadriceps tendon rupture [Article in German]. Oper Orthop Traumatol.

[27] Burnett RS, Berger RA, Della Valle CJ, Sporer SM, Jacobs JJ, Paprosky WG, Rosenberg AG (2005). Extensor mechanism allograft reconstruction after total knee arthroplasty. J Bone Joint Surg Am.

[28] Wise BT, Erens G, Pour AE, Bradbury TL, Roberson JR (2018). Long-term results of extensor mechanism reconstruction using Achilles tendon allograft after total knee arthroplasty. Int Orthop.

[29] Coladonato C, Hanna AJ, Patel NK (2024). Risk factors associated with poor outcomes after quadriceps tendon repair. Orthop J Sports Med.

